# Fabrication of Curcumin-Based Electrochemical Nanosensors for the Detection of Environmental Pollutants: 1,4-Dioxane and Hydrazine

**DOI:** 10.3390/bios14060291

**Published:** 2024-06-04

**Authors:** Renjith Kumar Rasal, Iffath Badsha, Muthaiah Shellaiah, Kumaran Subramanian, Abinaya Gayathri, Abdurahman Hajinur Hirad, Kumaravel Kaliaperumal, Thiyagarajan Devasena

**Affiliations:** 1 Centre for Nanoscience and Technology, Anna University, Chennai 600025, India; kumar.renjith12@gmail.com (R.K.R.); iffathbadsha@gmail.com (I.B.); 2 Department of Research and Analytics, Saveetha Dental College and Hospitals, Saveetha Institute of Medical and Technical Sciences (SIMATS), Saveetha University, Chennai 602105, India; muthaiahs.sdc@saveetha.com; 3 P. G. Research Department of Microbiology, Sri Sankara Arts and Science College (Autonomous), Kanchipuram 631561, India; kumarun23@gmail.com; 4 Unit of Marine Biomaterials and Natural Product Chemistry Research, Department of Orthodontics, Saveetha Dental College and Hospitals, Saveetha Institute of Medical and Technical Sciences (SIMATS), Saveetha University, Chennai 602105, India; abisumathi77@gmail.com; 5 Department of Botany and Microbiology, College of Science, King Saud University, P.O. Box 2455, Riyadh 11451, Saudi Arabia; ahirad@ksu.edu.sa

**Keywords:** MWCNT, curcumin, bisdemethoxycurcumin analog, hydrazine, 1,4-dioxane, electrochemical sensing

## Abstract

This work reports the development of novel curcuminoid-based electrochemical sensors for the detection of environmental pollutants from water. In this study, the first set of electrochemical experiments was carried out using curcumin-conjugated multi-walled carbon nanotubes (MWCNT–CM) for 1,4-dioxane detection. The MWCNT–CM/GCE showed good sensitivity (103.25 nA nM^−1^ cm^−2^ in the linear range 1 nM to 1 µM), with LOD of 35.71 pM and LOQ of 108.21 pM. The second set of electrochemical experiments was carried out with bisdemethoxy curcumin analog quantum dots (BDMCAQD) for hydrazine detection. The BDMCAQD/GCE exhibited good sensitivity (74.96 nA nM^−1^ cm^−2^ in the linear range 100 nM to 1 µM), with LOD of 10 nM and LOQ of 44.93 nM. Thus, this work will serve as a reference for the fabrication of metal-free electrochemical sensors using curcuminoids as the redox mediator for the enhanced detection of environmental pollutants.

## 1. Introduction

**Figure 1:**
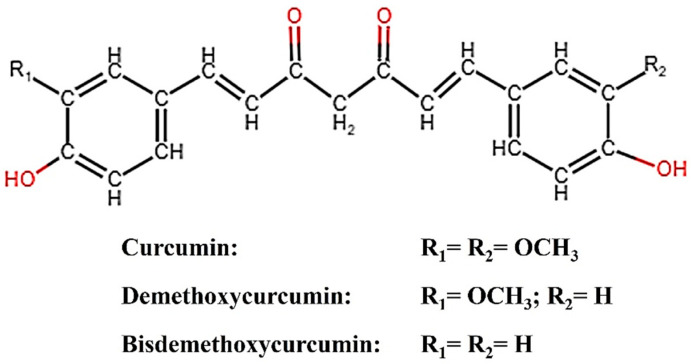
Structure of curcuminoids (CM, DMC, BDMC).

Plants have been utilized for a wide range of purposes since ancient times. One such plant is turmeric (*Curcuma longa*), the rhizome of which contains curcumin (CM), demethoxycurcumin (DMC), and bisdemethoxycurcumin (BDMC), broadly categorized as curcuminoids. Curcumin, the principal curcuminoid, possesses inherent electrocatalytic properties and has been reported to amplify the electrochemical response in sensor applications [1,2]. Structurally, the binding of CM to an analyte could be easily identified by spectroscopically analyzing the peaks corresponding to CM’s keto-enol moiety, phenolic, and methoxy group, leading to improved electrochemical response [3]. For enhanced sensing performance, CM conjugated with various carbonaceous nanostructures has been reported [1,2,3]. Figure 1 describes the structure of CM, DMC, and BDMC. Bisdemethoxy curcumin analog (BDMCA) is a highly stable structural analog of BDMC exhibiting antioxidant activities similar to CM [4,5]. The difference in the stability of BDMC and CM could be due to the absence of both the methoxy groups in BDMC which creates differences in the hydrogen-donating ability of the keto-enol moiety [5]. So far, there has been no report on the investigation of the electrocatalytic properties of BDMC. Since BDMC is present in trace amounts in turmeric, its structural analog was prepared and its electrochemical properties were explored for the first time in this research. 

CNTs are an incredible 1D allotrope of carbon, structurally composed of a hexagonally arranged matrix of sp^2^C atoms that impart unique characteristics, making them a profitable choice in a variety of electrochemical sensing applications [6,7]. CNTs are of relatively smaller size and possess a considerably larger surface area that is beneficial for the electrochemical sensing applications, offering good sensitivity, quick response, and remarkable reproducibility at ambient temperature [8]. When employed as an electrode material in electrochemical operations, the electronic characteristics of CNT imply that they could aid e^−^ mobility in the presence of electroactive species, facilitating improved electron transport in an aqueous medium [6,7,8].

1,4-Dioxane (C_4_H_8_O_2_) is an essential component in automotive fluids, antifreeze, and aircraft deicing procedures and is also utilized as a solvent in several industrial manufacturing systems [9,10]. It is released as a potentially harmful intermediate while producing ethylene glycol and ethylene oxide, which have been added to aviation gasoline for years as the standard antifreeze and deicers to prevent the freezing of petrol because of their low freezing point and low cost [11]. In the industrialized parts of the world, 1,4-dioxane-polluted water is reported to have surged, and industrial wastewater has become a predominant source of environmental pollution [12,13]. Furthermore, it pollutes the environment every single day through its use as a surfactant in cleansing and washing powder and also in the manufacturing of polyester [14,15]. Structurally, it is a heterocyclic di-ether molecule that manifests high resistance to degradation which further adds to its hazardous nature [13]. Its chemical structure enables it to persist in water bodies for an extended period due to its great aqueous miscibility, mobility, and stability in water, thereby contaminating surface and groundwater resources, disrupting the food chain, and accelerating the environmental damage [16,17,18,19]. The oral pathway is a common route of the potential risk for dioxane toxicity by consuming dioxane-contaminated water [20]. The WHO and other organizations of various countries have recommended a threshold limit of 0.56 µM dioxane concentration in drinking water in their recent guidelines [13]. So far, there are several conventional methods such as coagulation–precipitation, carbon adsorption, etc., for dioxane removal, but they are not efficient [21]. Many standard treatment methods utilizing hydrogen peroxide, ozone, and UV radiation help in removing dioxane but also pollute the environment secondarily [22]. It has also been reported that monooxygenase-producing bacteria can detect dioxane; however, this requires bacterial culture and its maintenance [23]. Sophisticated analytical techniques involving chromatographic and spectroscopic methods have been used for its detection [24,25]. Despite their high sensitivity, these techniques have limitations when it comes to trace level detection and also, they are time-consuming, expensive, and require trained personnel and complex sample preparation [26].

Hydrazine (N_2_H_4_) is a colorless liquid with density similar to that of water and is used as a rocket fuel propellant in high concentrations due to its high specific impulse. Based on a case report on the hydrazine leak from the emergency power unit (EPU) of F-16 aircraft, it was found that the inhalation exposure of even less than a minute to hydrazine, could spike the creatine phosphokinase (CPK) levels of healthy individuals who were 5 m away from the EPU. In addition to this, hydrazine has also been extensively used in several industrial and agricultural applications; however, its prolonged exposure makes it neurotoxic, carcinogenic, mutagenic, hepatotoxic, and nephrotoxic to humans and also pollutes the environment [27]. It is also reported that an increase in the hydrazine level beyond its critical standards could cause adverse effects, hence its level should be monitored [28,29,30]. Until now, fluorescence, chemiluminescence, chromatographic, spectroscopic, and electrochemical approaches have been used to detect N_2_H_4_ [31,32]. 

Out of all of the sensing techniques, electrochemistry is regarded as the most dependable and ideal technology, attributed to its greater sensitivity, affordability, eases of use, and time effectiveness. Nevertheless, the typical electrode materials which are frequently thought of as effective electrocatalysts for electrochemical detection are made of precious metals, the low abundance and high price of which have restricted their practical utility [20]. The electrochemical-sensing technique has recently gained popularity for its rapid detection of trace amounts of hazardous compounds in aqueous media. In addition to this, its excellent specificity, low cost, and quick response for detecting a wide range of analytes makes it an appealing method for analyzing the content of a sample by directly converting the response into an electrical signal [30,33]. Some reports suggest that by incorporating nanomaterials, the electrochemical response shows better sensitivity and limits of detection and improves their robustness. However, most of the nanoconjugates are metal-based inorganic catalysts that are expensive and not available easily. Since the discovery of graphitic-carbon-nitride-based electrocatalysts in 2009, a lot of research has been done on organic catalysts. Some of the recently reported curcumin-based electrochemical sensors are discussed as follows. Sana et al. 2022 developed a reduced graphene oxide–curcumin (rGO–CM) nanocomposite sensor for the electrochemical sensing of 1,4-dioxane. The rGO–CM sensor showed high sensitivity of 19.36 mA mM^−1^ cm^−2^ in the linear range 0.1 to 100 mM with 0.13 mM limit of detection (LOD) [34]. Iffath et al., 2023 reported the fabrication of a curcumin quantum dots (CMQD)-based electrochemical sensor for the ultrasensitive detection of dopamine. The CMQD sensor showed high sensitivity of 14.28 µA nM^−1^ cm^−2^ in the 0.05 nM to 1 nM linear range with 0.002 nM LOD [2]. Renjith et al., 2023 developed graphene oxide from curcumin and reported its electrochemical sensing performance towards 1,4-dioxane detection, with good sensitivity of 117 nA nM^−1^ cm^−2^ in the linear range 0.1 µM to 3 µM with 20.51 nM LOD [9]. In this present research, we developed two novel curcuminoid-based sensors to evaluate their potential to serve as redox mediators in the electrochemical sensing of environmental pollutants. The first set of electrochemical experiments was performed to investigate the electrochemical-sensing performance of MWCNT–CM-modified electrode towards 1,4-dioxane and the second set of electrochemical experiments was performed to investigate the electrochemical-sensing performance of BDMCAQD-modified electrode towards hydrazine. To the best of our knowledge, there has been no report on the investigation of the electrochemical properties of BDMCAQD. Hence, this research would set the foundation for further exploration of BDMCAQD in the field of electrochemistry for the sensing of pollutants and environmental remediation. 

## 2. Materials and Methods

### 2.1. Materials

Curcumin (C_21_H_20_O_6_), multi-walled carbon nanotubes (MWCNT Type 5), 1,4 dioxane (C_4_H_8_O_2_, 99%), acetonitrile (CH_3_CN, 99.5%), chlorobenzene (C_6_H_5_Cl, 99.8%), ethylene glycol (HOCH_2_CH_2_OH, 99.8%), potassium hexacyanoferrate (K_3_FeCN_6_), and phosphate buffer saline (PBS) were acquired from SRL Pvt. Ltd., Mumbai, India. Hydrazine hydrate and nafion solution were procured from Merck Pvt. Ltd., Mumbai, India. Sulphuric acid (H_2_SO_4_, 98%), nitric acid (HNO_3_, 70%), glacial acetic acid, acetylacetone, dimethyl formamide (DMF, 99.58%), salicylaldehyde, diethanolamine (DEA), and boric acid were purchased from SRL Pvt. Ltd., India. 

### 2.2. Apparatus

The morphological analysis of the prepared material was studied using scanning electron microscopy (Tescan, Vega.3.SBU, Kohoutovice, Czech Republic) and transmission electron microscopic imaging (TALOSF200S G2). ATR-FTIR spectroscopic analysis was performed using JASCO FT/IR-6600, Japan. The structural components of MWCNT–curcumin were examined and compared using the powder X-ray diffraction analysis (X’Pert Powder XRD System, Malvern, UK). The H1 NMR of MWCNT–curcumin was determined using an NMR spectrometer (Bruker NMR 400 MHz, Billerica, MA, USA). The synthesized BDMCA and BDMCAQDs and their maximum absorption spectra were recorded using UV-visible spectroscopy (T90+ PG Instruments, Lutterworth, UK), and the BDMCAQD emission spectra were recorded using photoluminescence (Pl) spectroscopy (FP 6500 Jasco, Tokyo, Japan). The electrochemical studies were carried out using the CH instrument 604E in ambient conditions. 

### 2.3. Synthesis of MWCNT–CM Conjugates

The production of MWCNT–CM conjugate was achieved following the previously adopted methodology [10]. In brief, MWCNT was functionalized to CM by mixing CM and MWCNT in a 10:1 ratio, subjecting them to a nitration process in the presence of H_2_SO_4_ and HNO_3_ mixture in the ratio 3:1 and heating at 60 °C, under constant stirring at 400 rpm for 12 h. This reaction mixture was incubated for 24 h at an ambient temperature of 28 °C. The obtained resultant after purification was characterized to ensure that the MWCNT–CM conjugate had formed. 

### 2.4. Synthesis of BDMCAQD

**Figure 2:**
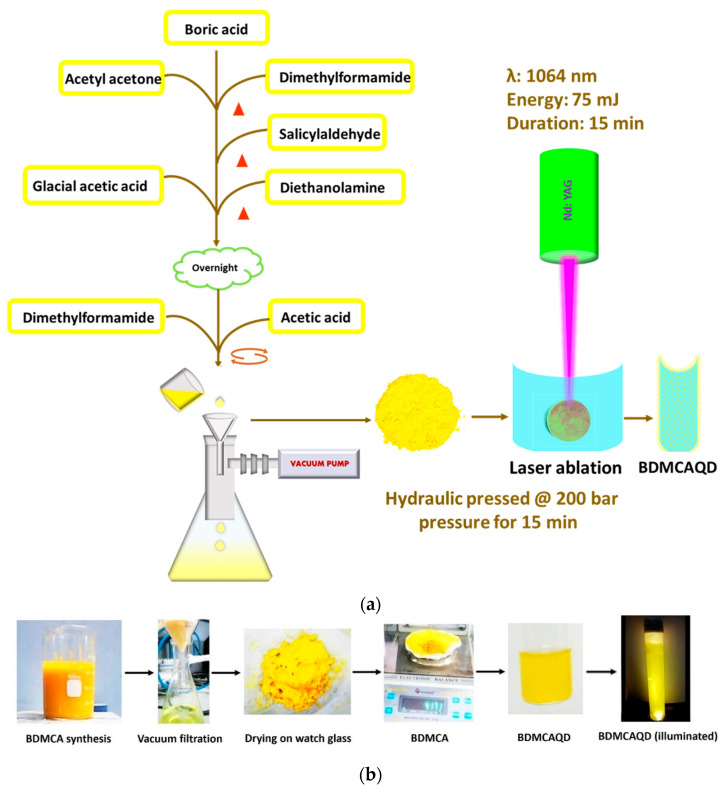
(**a**) Flow chart of the synthesis procedure of BDMCAQD. (**b**) Preparation of BDMCA and BDMCAQD.

A facile synthetical approach described earlier was employed for the synthesis of BDMCA [35]. Briefly, 5 g boric acid was added to a 2:1 ratio of acetylacetone and dimethylformamide (DMF) and heated for 15 min, to which a few mL of salicylaldehyde was dripped and again heated for 5 min. A solution formed of glacial acetic acid and diethanolamine was added to the above mixture and dried. To this, a few drops of DMF were added to form a fluid-like paste, which was then gradually added drop wise to 10% acetic acid under constant stirring. The mixture was then sonicated for 15 min, filtered, and dried. The obtained BDMCA was then converted to BDMCAQD by subjecting it to photo-oxidative plasma-induced irradiation process in which 75 mJ of 1064 nM wavelength infrared beam from neodymium-doped Yttrium Aluminium garnet radiation source was focused on a hydraulic-pressed BDMCA pellet (pressed at 200 bar for 15 min) [36]. Distilled water was utilized as the synthesis environment to form BDMCAQD because of its good heat capacity and minimal radiation absorption. The physical and electrochemical characteristics of the produced particles were then examined. Figure 2a depicts a flowchart of the BDMCAQD synthesis process. Figure 2b illustrates the phases in the synthesis process of BDMCAQD.

### 2.5. Electrode Preparation

A conventional 3 electrode system comprising glassy carbon electrode (GCE) as the working electrode having 0.0707 cm^2^ surface area, platinum wire as the counter electrode, and Ag/AgCl as the reference electrode. 0.1 M phosphate buffered saline (PBS) was used as the electrolyte. The working electrode was cleaned using alumina powder before the modification procedure. The working electrode was modified by coating it with 20 µL of 15 min ultrasonicated mixture comprising the prepared material (5 mg) in 0.5 mL ethanol and 10 µL of 0.5% Nafion solution by drop casting method. For BDMCAQD/GCE preparation, 50 µL of BDMCAQD was used to prepare the coating mixture. 

### 2.6. Electrochemical Studies

The electrochemical characterization of the prepared nanomaterial-coated GCE was performed using electrochemical impedance spectroscopy (EIS) and cyclic voltammetry (CV) analysis. The EIS experiments were carried out by dipping the three electrodes (working electrode, reference electrode, and counter electrode) in a 0.1 M phosphate-buffered saline (PBS) electrolyte containing 5 mM K_3_FeCN_6_, and the measurements were taken over a frequency range of 10 MHz to 100 KHz. The CV experiments were carried out in 0.1 M PBS electrolyte at a scan rate of 50 mVs^−1^ in a potential window of −1 to +1 V. The electrochemical sensing performance of MWCNT–CM/GCE towards 1,4 dioxane was done by adding different concentrations of 1,4-dioxane in 30 mL of 0.1 M PBS electrolyte at neutral pH and was analyzed by CV experiment in the potential window of −1 V to +1 V at the scan rate of 50 mV s^−1^. The electrochemical-sensing performance of BDMCAQD/GCE towards hydrazine was done by adding different concentrations of hydrazine in 30 mL of 0.1 M PBS electrolyte at neutral pH and was analyzed by LSV experiment at the scan rate of 50 mV s^−1^ in the potential window of 0 to +1.5 V. The limit of detection (LOD), limit of quantification (LOQ), and the sensitivity were calculated using the below Formulas (1), (2), and (3), respectively.

LOD = 3.3 σ/S(1)

LOQ = 10 σ/S(2)

Sensitivity = slope/electrode area (0.0707 cm^2^) (3)

σ—standard deviation of the y-intercept; S—slope of the calibration curve.

## 3. Results and Discussion

### 3.1. Mechanism of Formation of MWCNT–CM Conjugates

**Figure 3:**
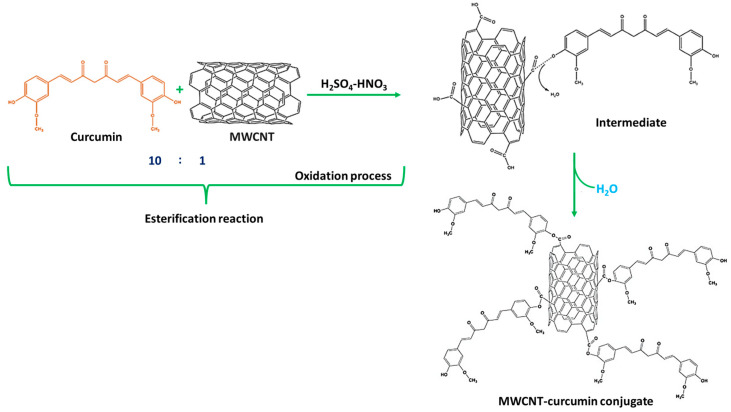
Schematic illustration of the mechanism of formation of MWCNT–curcumin.

The reaction of MWCNT and CM in the presence of H_2_SO_4_ and HNO_3_ was achieved in such a way that the esters were developed to alter the equilibrium. Ten parts of CM were utilized for every part of MWCNT. CM was employed in surplus since it is easier to remove CM than MWCNT during the purification of MWCNT–CM conjugate [37,38,39]. The implementation of HNO_3_ triggers the commencement of the oxidation process of MWCNT to oxidized MWCNT containing carboxyl (-COOH) functional groups [39,40,41]. The application of H_2_SO_4_ assists the catalysis process during oxidation, facilitating the esterification between the oxidized MWCNTs with CM forming MWCNT–CM conjugate [42]. Figure 3 depicts the mechanism of MWCNT–CM conjugate formation.

### 3.2. Mechanism of Formation of BDMCAQDs

**Figure 4:**
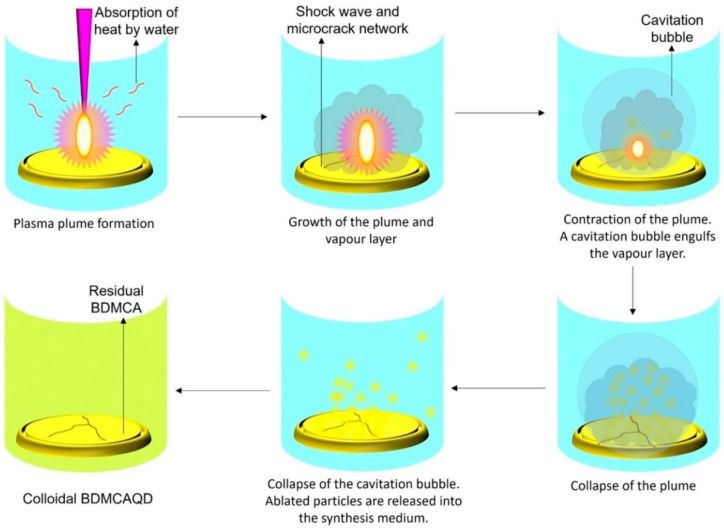
Mechanism of formation of BDMCAQD by laser ablation method.

The high energy irradiation beam produces photoinduced reactions of exothermic nature that drive the augmentation of the developed plasma plume at the interface between the hydraulically compacted BDMCA and the surrounding water resulting in a strong confinement effect. The heating up of the ambient water (as it absorbs heat from the radiation) and its subsequent evaporation due to the high pressure and temperature of the plasma, concocts liquid plasma that chemically reacts with the plasma plume causing the collapse of the vapor layer and cavitation bubble thereby dispersing ablated BDMCA particles into the surrounding water medium. The possible mechanism of formation of BDMCAQD is represented in Figure 4.

### 3.3. Physicochemical Characterization of MWCNT–CM Conjugates

**Figure 5:**
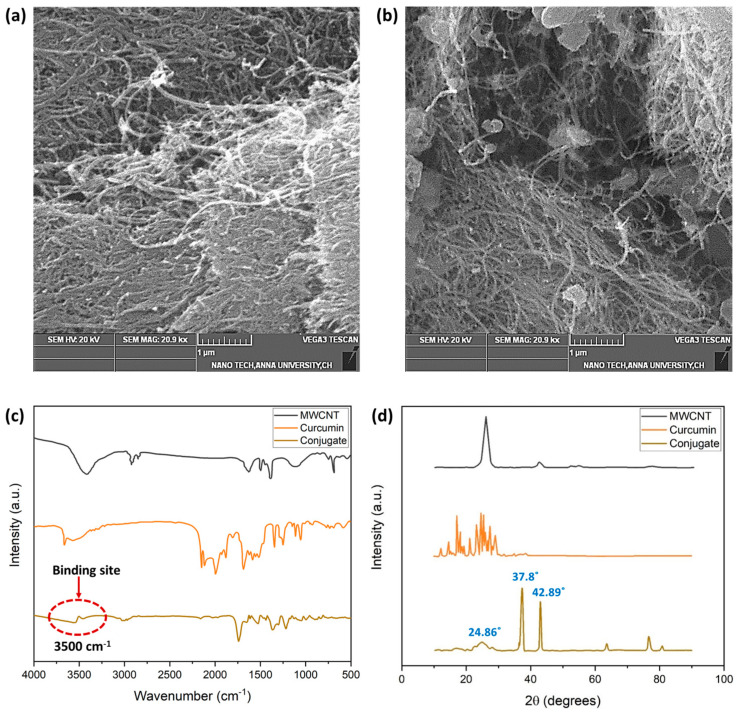
(**a**) SEM micrograph of MWCNT (1 µm scale) (**b**) SEM micrograph of MWCNT–curcumin conjugate (1 µm scale) (**c**) FTIR analysis of MWCNT, CM, and MWCNT–CM conjugate (**d**) XRD analysis of MWCNT, CM, and MWCNT–CM conjugate.

Figure 5 depicts the SEM images of the MWCNT and the MWCNT–CM. The shape of the MWCNT is a smooth, tubelike morphology with a high degree of aggregation, as shown in Figure 5a. The MWCNT–CM conjugate, on the other hand, shows the presence of particles attached to the nanotube’s surface (Figure 5b). Notably, decreased agglomeration was observed in MWCNT–CM as compared to pure MWCNT. The diminution in agglomeration engendered by functionalization exemplifies that CM is conjugated to the MWCNT [6].

The FTIR spectra (Figure 5c) provide insights into the structural functionalities and probable material interaction locations. The prominent bends and stretches of CM such as phenolic-OH stretch, benzene ring stretching, methyl C-H, C=O bonding, aromatic (C-O) stretch olefinic (C-H) bend, methyl -CH_3_ group, C-O-C stretch, benzoate (C-H vibration-trans), and aromatic ring (C-H) vibrations were observed at the wavenumbers 3503, 1594, 2847, 1496, 1429, 1271, 1149, 1023, 956, and 717, respectively [43]. It could be observed that the distinct peaks of MWCNT and CM diminish with a shift in the peak position in the MWCNT–CM conjugate. Based on the observations and previous reports, these structural changes at the molecular level could be considered as an indication of the formation of the MWCNT–CM conjugate. The drastic attenuation of the phenolic OH peak at 3500 cm^−1^ suggests that it might be the probable site of interaction of MWCNT with CM, which subsequently gets functionalized by forming a covalent bond between the phenolic –OH group of CM and carboxyl groups in the MWCNT. This aligns well with the earlier research findings [44].

MWCNT features a (002) reflection–diffraction peak, and the characteristic diffraction pattern of CM showing peaks between 2θ values 10° to 30° were attenuated in the MWCNT–CM conjugate (Figure 5d). The MWCNT–CM exhibited an intense peak at 2θ = 37.8°; a slightly shorter peak at 2θ = 42.89°; considerably low peaks at 62.92°, 77.23°, and 81.04°; and a broader peak at 2θ = 24.86°. Consequently, the observed peaks from the XRD pattern of the MWCNT–CM conjugate and the remarkable decline in the diffraction peaks’ intensity for the MWCNT–CM conjugate further support the formation of the MWCNT–CM conjugate [45,46,47,48,49].

### 3.4. Physicochemical Characterization of the BDMCAQD

**Figure 6:**
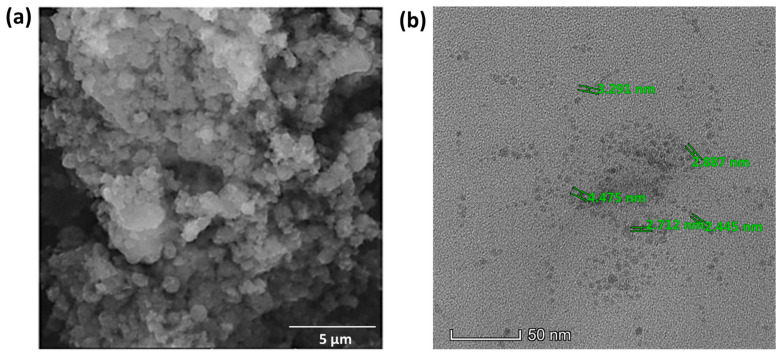
(**a**) SEM micrograph of BDMCA (5 µm scale) (**b**) TEM micrograph of BDMCAQD (50 nm scale).

**Figure 7:**
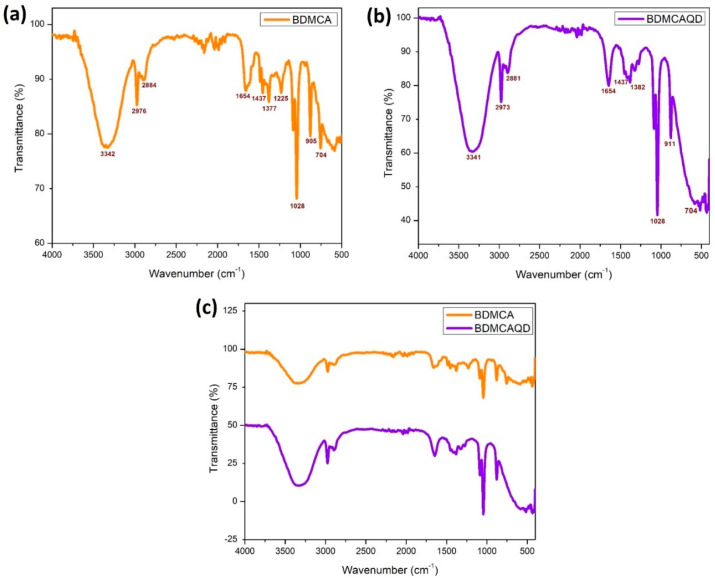
FTIR spectroscopy (**a**) BDMCA (**b**) BDMCAQD (**c**) BDMCA and BDMCAQD.

The SEM images of the synthesized BDMCA in Figure 6a show that BDMCA exists as irregularly shaped flakes. However, the TEM images of BDMCAQDs shown in Figure 6b show spherical dot-like morphology having an average size of around 3.16 nm ± 1.2. The FTIR analysis of BDMCA and BDMCAQD is represented in Figure 7. The prominent stretches and bends observed in the spectra are summarized in Table S1. Peaks about the benzene ring, alkene, ketone, and hydroxyl functional groups could be observed in the FTIR spectra of BDMCA and BDMCAQD. There were also observations of the O-H, aromatic C=C, C=O stretching, and C-H vibrations at the wavenumbers 3250–3650 cm^−^^1^, 1314–1654 cm^−^^1^, 1654 cm^−^^1^, 2344–2999 cm^−^^1^, respectively. The characteristic C=C stretch of this compound occurs at the frequency 1654–1437 cm^−1^. The vibrations at the frequency 704 cm^−^^1^ explain the band characteristics of the aromatic mono substitution. Collectively, the FTIR spectroscopic features confirm the presence of characteristic stretches and bends of BDMCA, such as aromatic rings, ortho hydroxy groups in the benzene rings, and conjugated carbon chains with unsaturated double bonds [35]. The similarities in the FTIR spectral peaks between BDMCA and BDMCAQD confirm that the integrity of the functional groups is maintained and that no structural changes occurred during the synthesis of BDMCAQD. The structure and the functional moieties present in BDMCA are responsible for the antioxidant activity of BDMCA, enabling it to act as a redox mediator. 

Thus, the size and functional group analysis of BDMCAQD indicates that the dots synthesized through the photoablation method are in a quantum regime and also retain the structural integrity of the native compound. Here, we justify that the protocol described is valid and optimal for synthesizing BDMCAQD. In addition, apart from the unique characteristic properties of BDMCA, these synthesized BDMCAQD would also offer more active sites for the electrocatalytic oxidation of the bound analyte leading to high-performance sensing of the analyte.

### 3.5. Electrochemical Characterization of MWCNT–CM Conjugates

**Figure 8:**
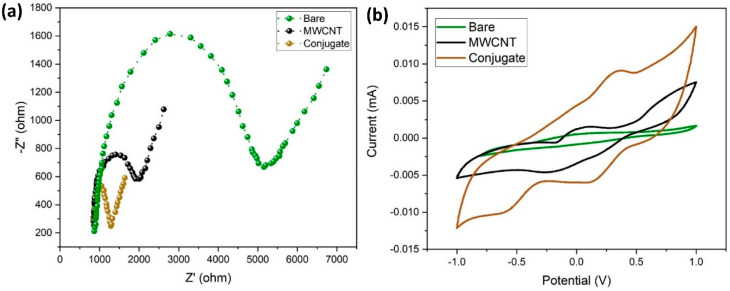
Electrochemical characterization of MWCNT–CM conjugate. (**a**) EIS plot. (**b**) CV plot.

In this study, EIS was used to measure the electrochemical impedances of bare GCE, MWCNT/GCE, and MWCNT–CM/GCE. The differences in the faradic impedances of these electrodes were recorded and shown in Figure 8a. The conjugate-modified electrode shows a relatively small charge transfer resistance (R_CT_), evident from being devoid of a semicircle in the EIS plot, indicating that it might be a suitable electrode material. The CV response of the bare GCE, MWCNT/GCE, and MWCNT–CM/GCE is shown in Figure 8b. Figure 8b shows no significant anodic (E_pa_) and cathodic (E_pc_) peak current response for the bare GCE; in addition, MWCNT/GCE exhibits E_pa_ at 0.056 V and E_pc_ at −0.221 V, and the MWCNT–CM/GCE exhibits E_pa_ at 0.355 V and E_pc_ at 0.112 V, indicating a notable shift of E_pc_ and E_pa_ for the MWCNT–CM-conjugate-modified electrode. The background current for the MWCNT–CM-conjugate-modified electrode is 9.08 times greater than the MWCNT-modified electrode. The increase in current and shift in voltage could be attributed to the electrocatalytic behavior of curcumin present in the conjugate.

### 3.6. Electrochemical Sensing Performance of the MWCNT–CM Conjugate towards 1,4-Dioxane

**Figure 9:**
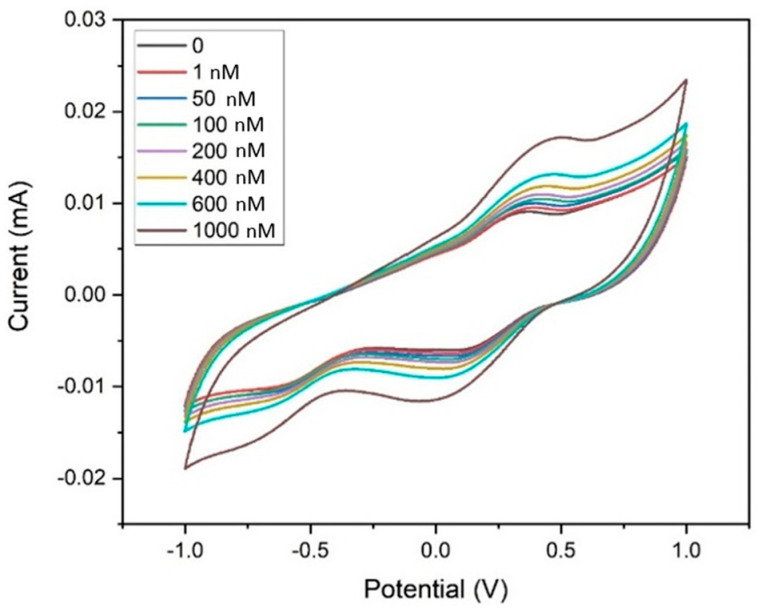
Cyclic voltammogram of the MWCNT–CM-conjugate-modified GCE towards 1,4 dioxane (1 nM to 1 µM).

**Figure 10:**
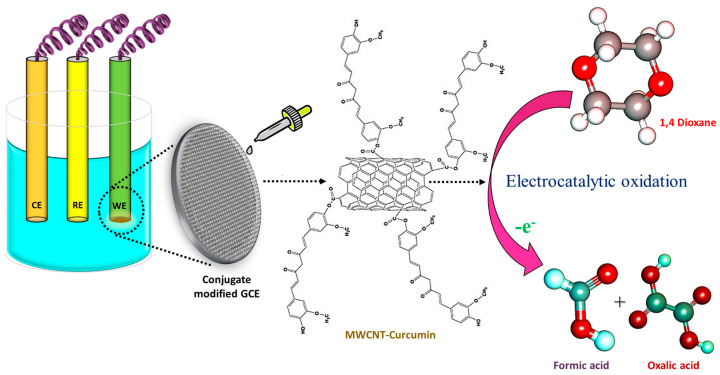
Sensing mechanism of 1,4-dioxane by MWCNT–CM-modified electrode.

Figure 9 shows the electrochemical sensing performance of MWCNT–CM/GCE towards 1,4-dioxane in the detection range of 1 nm to 1 µM by CV experiments. The current response during each rise in dioxane concentration indicated that when dioxane was not present, the E_pa_ appeared at 0.355 V, but with the addition of 1 nM of 1,4-dioxane, the peak shifted to 0.45 V. A consistent rise in current at 0.45 V was likewise seen when the dioxane concentration increased from 1 nM to 1 µM. The associated calibration curve, which shows the concentration (nM) vs. the measured peak current (µA) was generated (Figure S1) to obtain the regression (linear) equation which was computed to be I(µA) = 0.0073x (nM) + 9.4853 showing R^2^ = 0.982. The limit of detection (LOD) and the limit of quantification (LOQ) were calculated to be 35.71 pM and 108.21 pM, respectively, and the sensitivity was found to be 103.25 nA nM^−^^1^ cm^−^^2^ in the linear spectrum 1 nM to 1 µM. It was observed from Figure 10 that the peak current increases upon the addition of 1,4-dioxane. Hence, it could be inferred that the pH does not affect the electrode’s performance. As 1,4-dioxane is a strong base, its addition to the electrolyte changes the pH of the electrolyte from neutral to basic.

### 3.7. Possible Sensing Mechanism of 1,4-Dioxane by MWCNT–CM/GCE

Earlier studies involving 1,4-dioxane sensors hypothesized several oxidation pathways. Based on the shift in pH during the electrocatalytic oxidation of dioxane, some researchers described the emergence of acidic intermediates during the breakdown of 1,4-dioxane that could donate electrons to electron acceptors like aromatic rings. Studies have also witnessed that under appropriate applied potential, 1,4-dioxane could well be oxidized in neutral pH. The incorporation of curcumin facilitates the oxidation kinetics via its electron-drawing methoxy group, thereby aiding in the oxidation of 1,4-dioxane (Figure 10). In addition to this, curcumin present in the conjugate increases the electrode’s affinity for the target analyte. The electrons released during the redox process contribute to improving the sensing medium’s conductivity. Consequently, as the amount of dioxane increases, the electrochemical response of the system also increases linearly [9].

### 3.8. Interference Study

**Figure 11:**
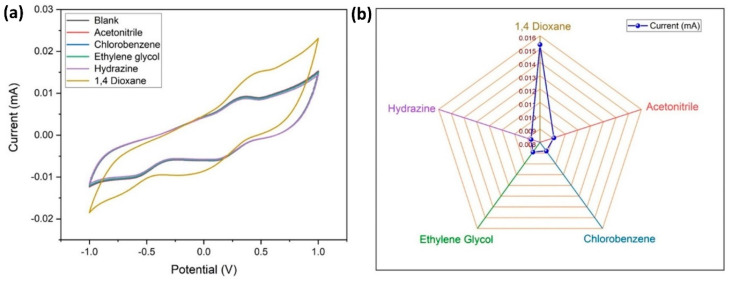
Specificity of the conjugate-modified GCE towards 1,4-dioxane. (**a**) Cyclic voltammogram of the current response indicating the specificity of the conjugate-modified GCE towards 1,4-dioxane. (**b**) Comparison of the peak current values for 1,4-dioxane and its interferents.

It is necessary to check the specificity of the electrode to the target analyte (1,4-dioxane), therefore an electrochemical study was carried out using CV (at similar conditions described earlier) with interfering materials like acetonitrile, chlorobenzene, ethylene glycol, and hydrazine, which are frequently used solvents in aerospace applications (Figure 11a,b). It is apparent that there is no visible variation in the response current for the interring materials, whereas there is a considerable variation in E_pa_ and E_pc_ for 1,4 dioxane. From this, it is confirmed that the MWCNT–CM is specific towards 1,4 dioxane and can be used as a 1,4 dioxane sensor.

### 3.9. Reliability and Stability of the MWCNT–CM Sensor 

The reliability of the presented sensor was validated by repeatedly executing 50 CV runs in 30 mL of 0.1 M PBS with 1 µM dioxane since reliability is an important criterion for practical applications. For the first 35 runs, (RSD = 1.31%). Figure S2a shows no discernible change in peak current. Only after the 35th run, the current responsiveness decreases gradually with each subsequent run, reaching a sharp peak after the 40th run. By recording the E_pa_ of the modified electrode in 1 µM dioxane in electrolyte for 12 weeks, the stability of this suggested sensor was evaluated. The obtained results are presented in Figure S2b which shows no visible variation in E_pa_ for the first seven weeks (RSD = 1.56%), following which a gradual decline in the E_pa_ was observed.

### 3.10. Reproducibility of MWCNT–CM Sensor

The reproducibility of the MWCNT–CM /GCE sensor was studied by performing CV experiments using three different MWCNT–CM-coated GCEs with 500 Nm concentration of 1,4-dioxane at 50 mVs^−1^ scan rate, −1 to +1 V potential window, and PBS (0.1 M), and the results are shown in Figure S3. From Figure S3, it could be inferred that there is no significant variation in the current response for all of the 3 different MWCNT–CM-coated GCE electrodes (RSD = 1.3%, n = 3). This shows that the proposed sensor possesses good reproducibility.

### 3.11. Electrochemical Characterization of BDMCAQD

**Figure 12:**
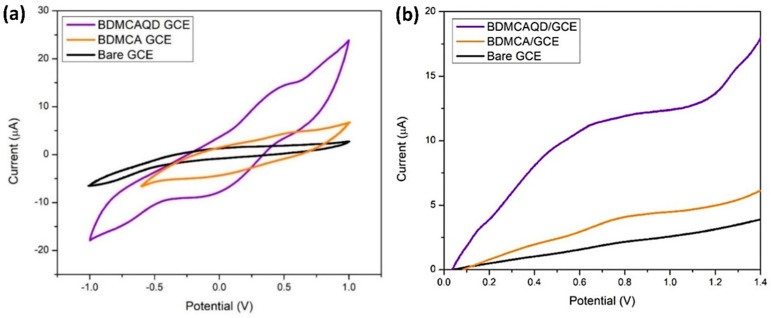
Electrochemical response (**a**) CV response (**b**) LSV plot.

From Figure 12a, it is obvious that the bare GCE lacks a distinct oxidation and reduction peak, while oxidation and reduction peaks were observed for BDMCA and BDMCAQD-modified electrodes. The E_pa_ and E_pc_ for both the BDMCAQD- and BDMCA-modified GCEs were observed at 0.55 V and −0.18 V, respectively. However, the E_pa_ (13.82 µA) of BDMCAQD-modified GCE was 3.2 times higher than the E_pa_ of BDMCA- (3.63 µA) modified GCE. This increase in the current response is because of more active sites on the BDMCAQD. Similarly, Figure 12b further confirms the results obtained from CV showing a 3.8 times higher anodic peak current for BDMCAQD- than BDMCA-modified electrodes.

### 3.12. The Electrochemical Sensing Performance of the BDMCAQD towards Hydrazine

To analyze the electrochemical response of BDMCAQD-modified electrodes towards hydrazine, LSV was performed at similar conditions to those described earlier, with 1 µM hydrazine in the electrolyte. From Figure S4, a significant peak shift from 0.55 V to 0.65 V is observed when hydrazine was introduced, and the E_pa_ current response also showed a significant increase, which is due to the redox reactions occurring between BDMCAQD with hydrazine.

**Figure 13:**
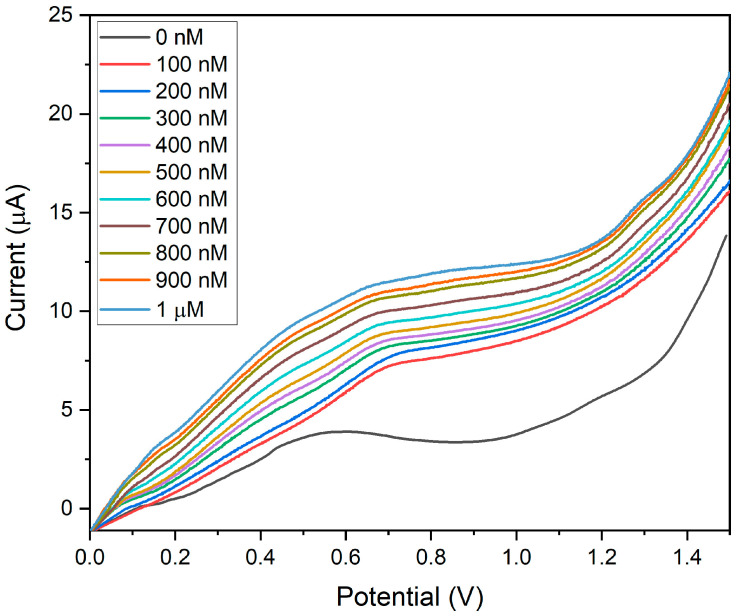
LSV plot showing the sensitivity of BDMCAQD/GCE towards hydrazine (100 nM to 1 µM).

The sensitivity of BDMCAQD/GCE was validated by performing electrochemical studies to sense the varying concentrations of hydrazine (100 nM to 1 µM) utilizing the LSV approach. The BDMCAQD-modified electrode was stabilized in the electrolyte at neutral pH before the addition of the analyte. The response current for every increase in analyte concentration is shown in Figure 13, which shows that the E_pa_ occurs at 0.55 V when hydrazine is not present, but shifts to higher potential (0.65 V) with the introduction of the analyte (100 nM hydrazine). The LSV curve showed a steady rise in current as the analyte (N_2_H_4_) concentration increased from 100 nM to 1 µM. The developed electrode for hydrazine sensing at very low levels has a linear correlation coefficient, as this study highlights. The resulting calibration curve is displayed in Figure S5, where the concentration (nM) is plotted against the measured current response (µA).

The linear regression equation was derived from the calibration plot as I (µA) = 0.0053x + 6.07 with R^2^ = 0.99. The LOD was calculated to be 10 nM, and the LOQ was found to be 44.93 nM. The sensitivity was determined to be 74.96 nA nM^−1^ cm^−2^ for the linear spectrum 100 nM to 1 µM, which is in alliance with the threshold standards of hydrazine in drinking water, and that is below 312 nM (threshold limit described by USEPA). From Figure 13, it could also be inferred that the pH does not affect the electrode’s performance, as evident from the increase in peak current with the rise in the concentration of hydrazine which is basic in nature.

The electrochemical sensing parameters obtained for MWCNT–CM/GCE and BDMCAQD/GCE were compared with the reported literature and are tabulated in Tables S2 and S3, respectively. The comparison shows that the sensitivity, LOD, and LOQ exhibited by both MWCNT–CM and BDMCAQD are on par with the recently reported literature.

### 3.13. Possible Sensing Mechanism of Hydrazine by BDMCAQD/GCE

**Figure 14:**
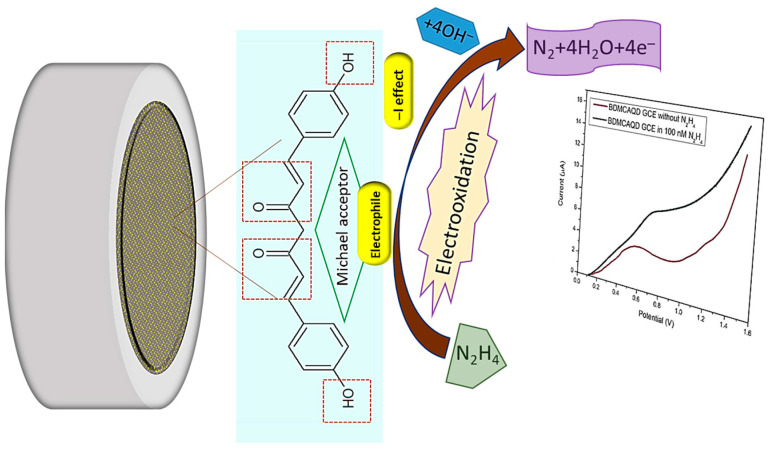
Possible sensing mechanism of BDMCAQD towards hydrazine.

The electrocatalytic effect of inherently electroactive BDMCAQD could be attributed to its physical properties and chemical structure. Based on the FTIR analysis, the ortho-hydroxyl group of the aromatic ring structure acts as an electron-withdrawing group by inductive (-I) effect, and the α, β-unsaturated carbonyl group exhibiting Michael accepting features acts as an electrophile, facilitating the oxidation of hydrazine to nitrogen. In addition to this, the size reduction to the quantum regime confers more active sites for analyte binding with BDMCAQD. All these characteristic features of BDMCAQDs enhance the electrochemical performance of the proposed novel sensor towards hydrazine. The possible sensing mechanism is pictorially represented in Figure 14 [2].

### 3.14. Interference Study

**Figure 15:**
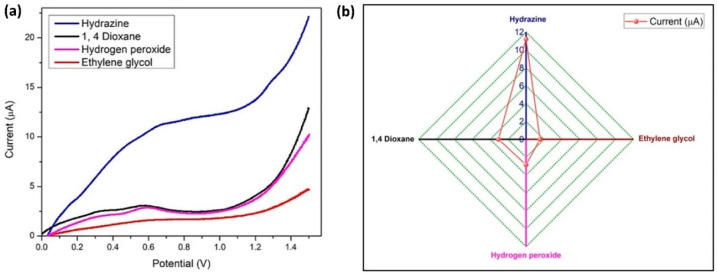
Specificity of BDMCAQD-modified electrode towards hydrazine (**a**) LSV plot of the current response indicating the specificity of BDMCAQD towards hydrazine; (**b**) Comparison of the peak current values for hydrazine and its interferents.

The LSV method was used for running a sensitivity test with 100 nM hydrazine at similar conditions, with 1 µM concentration of three interfering materials (hydrogen peroxide, ethylene glycol, and 1,4-dioxane) which are frequently used chemicals in the aircraft industry as propellants and deicing solvents. Four anodic oxidation peaks were visible in Figure 15a, and hydrazine exhibited the largest current response. A comparison graph of the current (peak) response values for hydrazine and its interferents is presented in Figure 15b. Hydrazine has a different oxidation potential than other interferents, which indicates that BDMCAQD/GCE is quite selective for hydrazine.

### 3.15. Reliability and Stability of BDMCAQD Sensor

The reliability of the BDMCAQD-modified GCE sensor was determined by performing 20 LSV runs in 1 nM hydrazine. For the first 15 runs, the peak current did not change significantly, having a relative standard deviation (RSD) of 0.38, as can be shown in Figure S6a. Peak current gradually reduced after the 15th run, because of the deposition of oxidation products on the electrode’s surface, which eventually blocks the active sites needed for hydrazine binding. The stability of BDMCAQD/GCE was determined by recording the response current of 1 nM hydrazine for seven weeks of the modified electrode and continuing the same electrode for seven weeks (Figure S6b). Following each run, the modified electrode was kept in a vacuum desiccator at room temperature. The LSV’s current response did not significantly vary for the first five weeks. After the fifth week, a marginal decline in the peak current was seen with an RSD of 0.31%, proving the stability of the sensor.

### 3.16. Reproducibility of BDMCAQD Sensor

The reproducibility of the BDMCAQD/GCE sensor was studied by performing LSV experiments using three different BDMCAQD-coated GCEs with 500 nM hydrazine concentration at 50 mVs^−1^ scan rate, 0 to +1.4 V potential window, and PBS (0.1 M), and the results are shown in Figure S7. From Figure S7, it could be inferred that there is no significant variation in the current response for all of the 3 different BDMCAQD-coated GCE electrodes (RSD = 2.1%, n = 3). This shows that the proposed sensor possesses good reproducibility.

## 4. Real-Time Analysis

The real-time analysis of the prepared sensor was performed by adding the respective analyte (500 nM) to the drinking water and tap water sample. Before performing the analysis, the electrodes were stabilized in PBS for 30 min. The added and measured concentrations of the respective analytes are shown in Table S2, from which the recovery rate was calculated. The MWCNT–CM/GCE sensor showed a recovery rate of 98.4% and 93.6% in drinking and tap water samples, respectively. Likewise, The BDMCAQD/GCE sensor showed a recovery rate of 99.4% and 95.6% in drinking and tap water samples, respectively. This experiment proves that both MWCNT–CM/GCE and BDMCAQD/GCE are suitable for the detection of 1,4-dioxane and hydrazine in real water samples, respectively. 

## 5. Conclusions

Overall, successful modification of the glassy carbon electrode to form MWCNT–CM/GCE and BDMCAQD/GCE was done and used for the detection of 1,4-dioxane and hydrazine, respectively. MWCNT and CM were conjugated by a simple chemical esterification process. The MWCNT–CM/GCE showed an excellent specificity towards 1,4-dioxane detection, exhibiting a sensitivity of 103.25 nA nM^−1^ cm^−2^ for linear range of 1 nM to 1 µM, having a LOD of 35.71 pM and LOQ of 108.21 pM. BDMCAQDs were prepared by the laser ablation method, and its electrocatalytic performance was investigated for the first time in this research. BDMCAQD/GCE exhibited good electrochemical response with high specificity towards hydrazine, and excellent sensitivity of 74.96 nA nM^−1^ cm^−2^ in the linear range 100 nM to 1 µM with a LOD of 10 nM and LOQ of 44.93 nM. The remarkable sensing performance of both MWCNT–CM/GCE and BDMCAQD/GCE proves that both MWCNT–CM and BDMCAQD could effectively serve as good electrode material for the electrochemical sensing of 1,4-dioxane and hydrazine, respectively. This research thus lays the foundation for the exploration of the electrochemical applications of curcuminoids for environmental applications. 

## Data Availability

All the data associated with this manuscript are provided in the article itself.

## Supplementary Materials

The following supporting information can be downloaded at: https://www.mdpi.com/article/10.3390/bios14060291/s1.
