# Supplementary material for: Fabrication of Curcumin-Based Electrochemical Nanosensors for the Detection of Environmental Pollutants: 1,4-Dioxane and Hydrazine
Source: Biosensors (Basel). 2024 Jun 4;14(6):291. doi: 10.3390/bios14060291 (PMC11202126; doi:10.3390/bios14060291)
Supplement: Supplementary file 1 [file biosensors-14-00291-s001.zip › biosensors-2974160-supplementary.pdf]

SUPPLEMENTARY DATA

SUPPLEMENTARY FIGURES

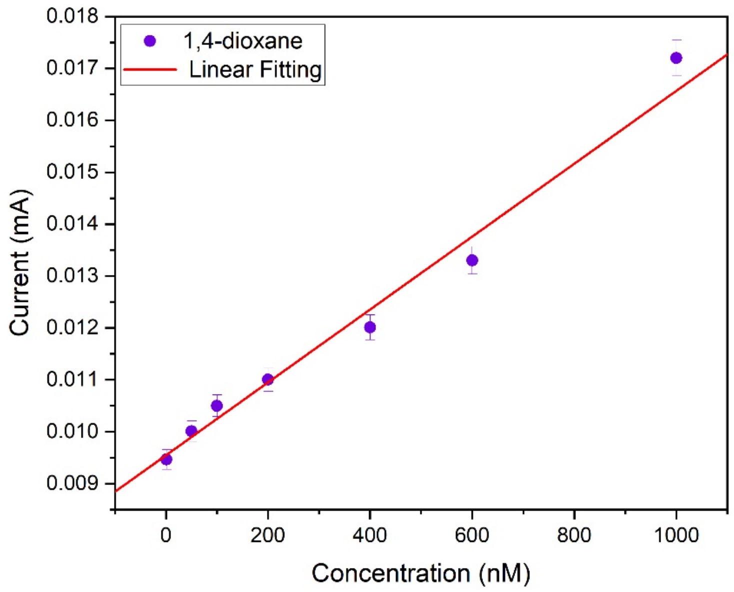

Figure S1. Linear plot of oxidation current versus concentrations of 1,4 dioxane.

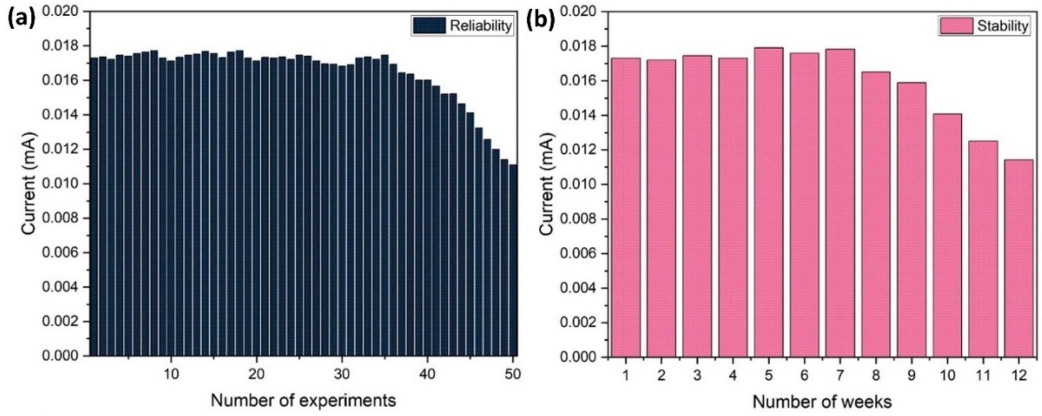

Figure S2. (a) Reliability of MWCNT-CM/GCE as 1,4-dioxane sensor (b) Stability of WCNT-CM/GCE as 1,4-dioxane sensor.

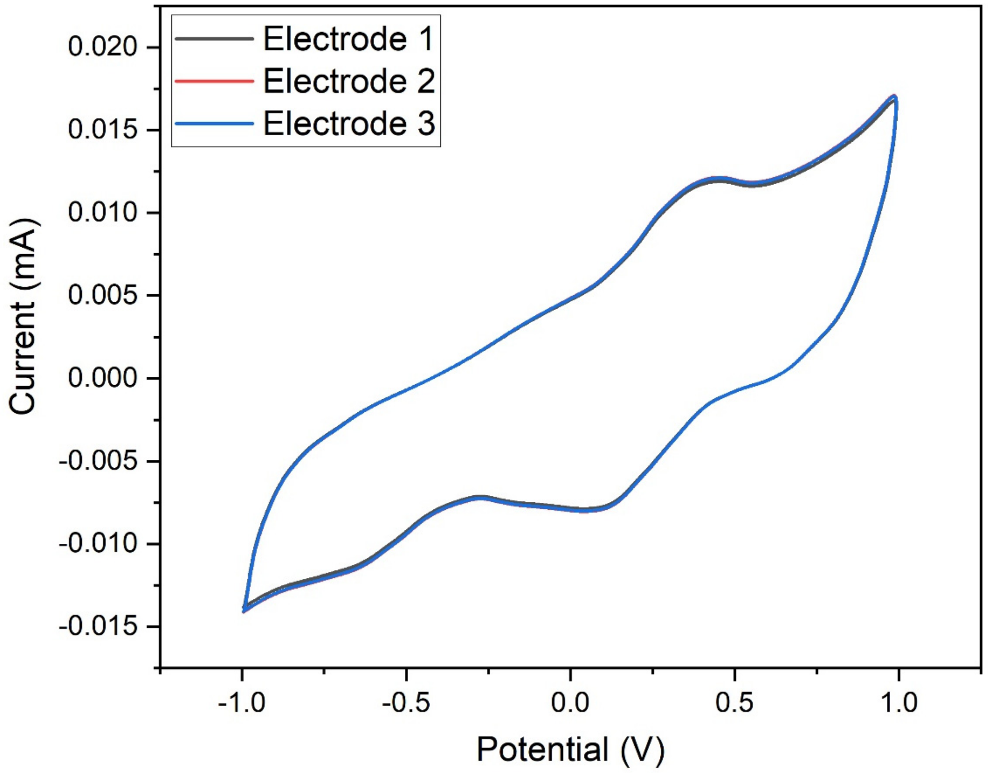

Figure S3. Reproducibility of MWCNT-CM sensor.

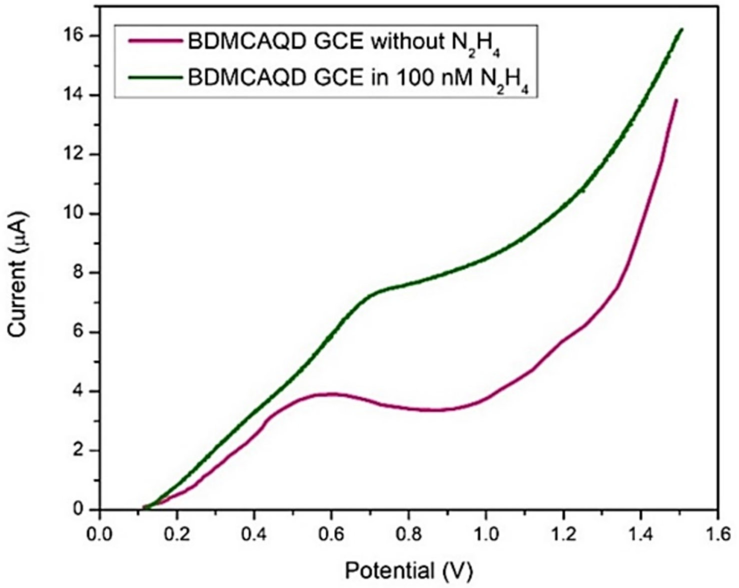

Figure S4. Comparative assessment of voltage response of BDMCAQD modified electrode with and without hydrazine.

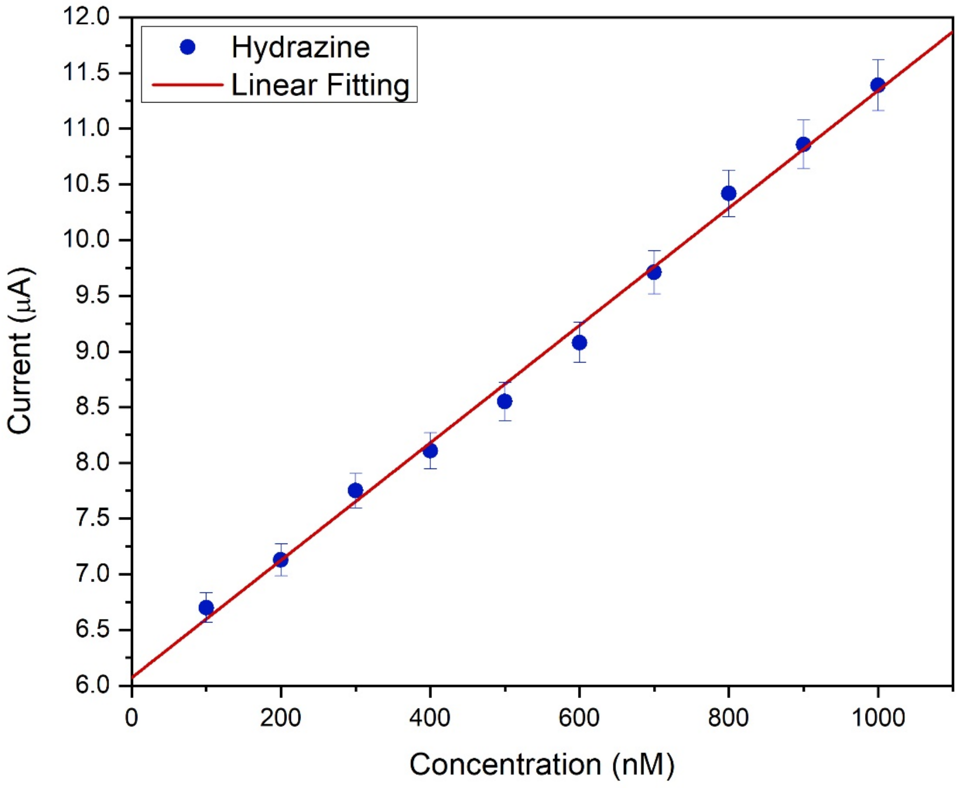

Figure S5. Linearity graph for the current response vs analyte concentrations.

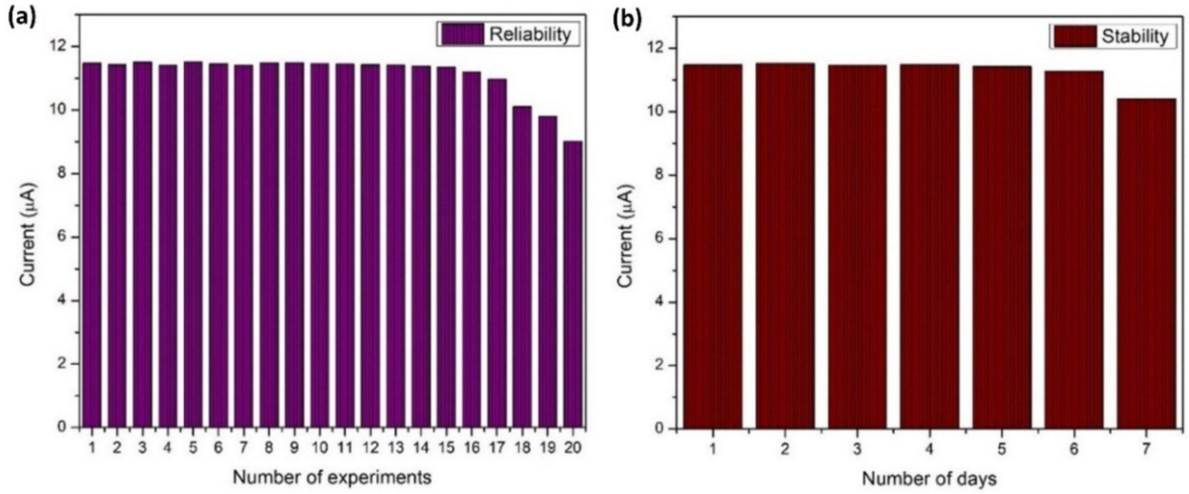

Figure S6. Sensor performances (a) Reliability of BDMCAQD/GCE as hydrazine sensor (b) Stability of BDMCAQD/GCE as hydrazine sensor.

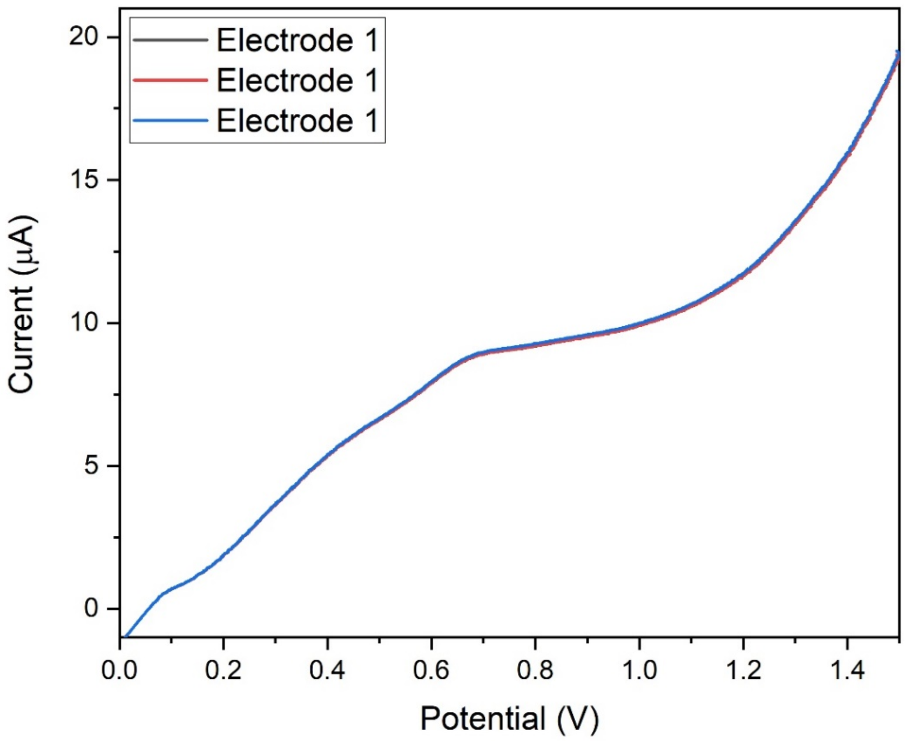

Figure S7. Reproducibility of BDMCAQD sensor.

SUPPLEMENTARY TABLES

Table S1. Interpretation of the functional groups present in BDMCA and BDMCAQD.

Frequency range (cm^{-1})	Functional groups	Bonds in BDMCA and BDMCAQD	Interpretation
3600–3300	Phenolic OH stretch	O-H at ortho position	O-H stretch
2999–2344	Methylene (symmetric/ asymmetric) group	C-H in the conjunction chain	C-H stretch
1654–1314	Alkenyl C-C conjugated ketone	Ketone in the linear chain	C=O olefinic stretch
1437	Aromatic ring stretches	Aryl C-C bond	C-C stretch denotes the benzene ring
1028-900	Aromatic bend-out of plane	Skeletal C-C bond vibrations	C-C stretch denotes the conjugated chain
704	Substitution at the ortho position	Phenolic (C-O-H) stretch	Presence of ortho hydroxyl group

Table S2. Comparison of 1,4-dioxane detection using MWCNT-curcumin modified electrode with other reported studies.

S. No.	Electrode material	Technique	Linear range (μM)	Sensitivity (μA μM $^{-1}$ cm^{-2})	LOD (nM)	References
1.	**Reduced graphene oxide-curcumin nanocomposite**	Linear Sweep Voltammetry	100 to 10000	19.36	130	[34]
2.	**ZnO/GO nanocomposite**	Linear Sweep Voltammetry	0.0001 to 10	7.6899	0.012	[36]
3.	**ZnO/ZiO/MnO$_2$ nanocomposite**	Linear Sweep Voltammetry	0.00012 to 1200	1.0417	0.009	[20]
4.	**NiO doped Nd$_2$O$_3$**	Linear Sweep Voltammetry	0.00012 to 1200	0.029	0.033	[14]
5.	**Graphene oxide nanosheets derived from curcumin**	Linear Sweep Voltammetry	0.1 to 3	117	20.51	[9]
6.	**MWCNT-CM conjugate**	Cyclic Voltammetry	0.001 to 1	103.25	0.035	This work

Table S3. Comparison of the proposed BDMCAQD sensor with recently published sensing systems for hydrazine detection.

S. No.	Electrode material	Technique	Linear range (µM)	Sensitivity (µA µM $^{-1}$ cm^{-2})	LOD (nM)	References
1.	**Au-modified the hollow $ZnSnO_3$ cubes**	Cyclic Voltammetry	0.02 to 360	0.787	10	[32]
2.	**Fluorinated cobalt phthalocyanine**	Cyclic Voltammetry	1 to 370	0.132	200	[37]
3.	**MoS_2/WO_3 hybrid composite**	Linear Sweep Voltammetry	5 to 200	0.121	3930	[38]
4.	**Biomass-derived carbon quantum dots**	Chronoamperometry	125 to 1125	0.151	39700	[45]
5.	**$NiCo_2S_4$ nanocomposite**	Chronoamperometry	1.7 to 7800	0.179	600	[47]
6.	**BDMCAQD**	Linear Sweep Voltammetry	0.1 to 1	74.96	10	This work

Table S4. Real time analysis for both MWCNT-CM and BDMCAQD sensor

Sensor	Analyte concentration		
	**Analyte**	**Drinking Water**	**Tap Water**
**MWCNT-CM/GCE**	1,4-dioxane- Added	500 nM	500 nM
	1,4-dioxane- Measured	492 nM	468 nM
	Recovery Rate	98.4%	93.6%
**BDMCAQD/GCE**	Hydrazine- Added	500 nM	500 nM
	Hydrazine- Measured	497 nM	478 nM
	Recovery Rate	99.4%	95.6%
